# Fully Automated Computational Approach for Precisely Measuring Organelle Acidification with Optical pH Sensors

**DOI:** 10.1021/acsami.2c00389

**Published:** 2022-04-11

**Authors:** Anil Chandra, Saumya Prasad, Francesco Alemanno, Maria De Luca, Riccardo Rizzo, Roberta Romano, Giuseppe Gigli, Cecilia Bucci, Adriano Barra, Loretta L. del Mercato

**Affiliations:** † Institute of Nanotechnology, National Research Council (CNR-NANOTEC), Campus Ecotekne, Via Monteroni, Lecce 73100, Italy; ‡ Dipartimento di Matematica e Fisica, Università del Salento, Via Monteroni, Lecce 73100, Italy; § Dipartimento di Scienze e Tecnologie Biologiche ed Ambientali (DiSTeBa), Università del Salento, Via Monteroni, Lecce 73100, Italy; ∥ Istituto Nazionale di Fisica Nucleare, Sezione di Lecce, Via Monteroni, Lecce 73100, Italy

**Keywords:** ratiometric pH sensors, silica microparticles, fluorescence, pH sensing, organelle acidification, microparticle tracking, data compression, automated cluster analysis

## Abstract

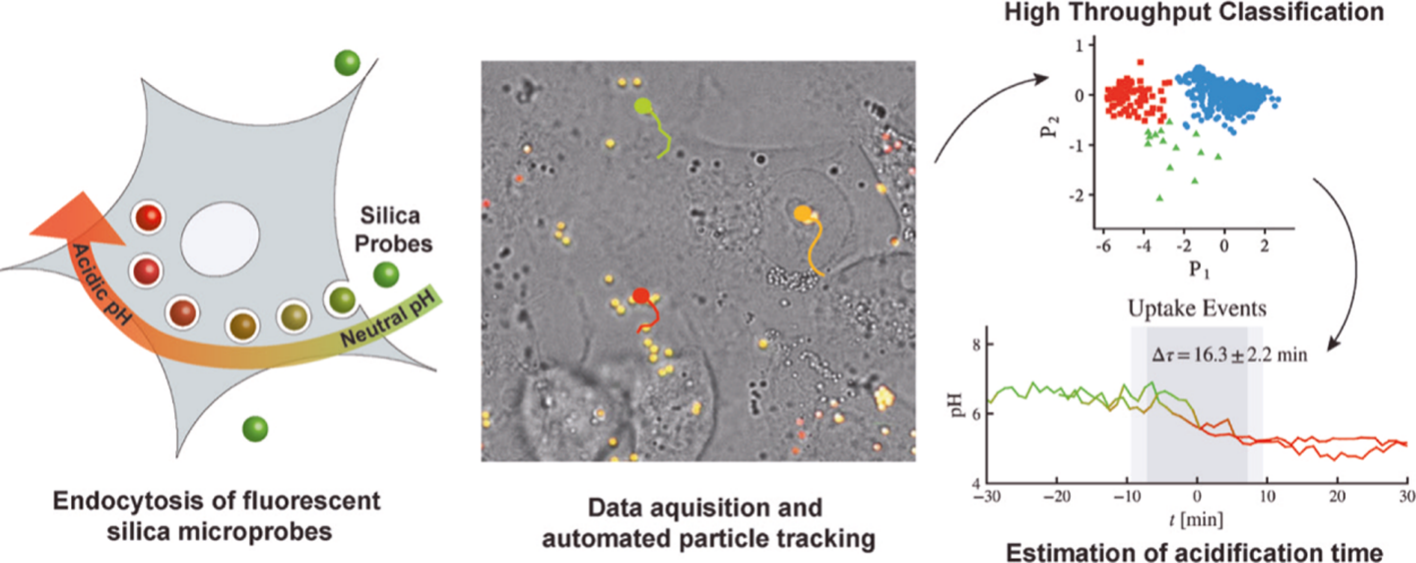

pH balance and regulation within organelles are fundamental to cell homeostasis and proliferation. The ability to track pH in cells becomes significantly important to understand these processes in detail. Fluorescent sensors based on micro- and nanoparticles have been applied to measure intracellular pH; however, an accurate methodology to precisely monitor acidification kinetics of organelles in living cells has not been established, limiting the scope of this class of sensors. Here, silica-based fluorescent microparticles were utilized to probe the pH of intracellular organelles in MDA-MB-231 and MCF-7 breast cancer cells. In addition to the robust, ratiometric, trackable, and bioinert pH sensors, we developed a novel dimensionality reduction algorithm to automatically track and screen massive internalization events of pH sensors. We found that the mean acidification time is comparable among the two cell lines (Δ*T*_MCF-7_ = 16.3 min; Δ*T*_MDA-MB-231_ = 19.5 min); however, MCF-7 cells showed a much broader heterogeneity in comparison to MDA-MB-231 cells. The use of pH sensors and ratiometric imaging of living cells in combination with a novel computational approach allow analysis of thousands of events in a computationally inexpensive and faster way than the standard routes. The reported methodology can potentially be used to monitor pH as well as several other parameters associated with endocytosis.

## Introduction

Gradual acidification of endosomes is a fundamental step during the endocytic pathway, where the appropriate pH of endosomes and lysosomes at different time points is an important factor governing the fate of the endosome and the endocytosed material.^1−6^ An example of importance of pH in endosomes is involvement of acidic pH in activating lysosomal acid hydrolases that are involved in breakdown of nucleic acid, protein, sugar, and lipid to be utilized by the cell as building blocks. This lysosomal acidity is generated and maintained by the activity of the vacuolar ATPase (V-ATPase) that pumps protons into the lumen of endosomes and lysosomes.^7−13^

Classically, the endocytic pathway was defined and considered as the pathway to internalize and process materials from the extracellular milieu. Being involved in delivering nutrients to the cells, nutrient sensing, and catabolism for receptor recycling, endocytosis is also important for internalization and killing of infective agents and to terminate cell signaling. The ways by which endocytic pathways can impact life of a cell are very diverse, starting from the fact that endosomes and lysosomes control cell signaling and regulate apoptosis, cell migration, autophagy, and many more cellular processes through modulation of endocytosis.^14,15^ Because of these array of effects on different cellular processes, alterations of the endocytic pathway has been linked with numerous human diseases where a particularly crucial step seems to be acidification. For instance, defects in the acidification of endocytic organelles, such as endosomes and lysosomes, contribute significantly to pathogenesis in lysosomal storage disorders, neurodegenerative diseases, autoimmune diseases, and infectious diseases in addition to tumor formation and dissemination.^16,17^ Therefore, it is of interest to study the phenomena of acidification to unravel different molecular mechanisms controlling this process. Monitoring acidification over time may help in developing strategies to counteract dysfunctions and thus find therapies for several human diseases. This is particularly important for cancer, where altered acidification is an early event that is now considered a hallmark of cancer and fundamental for cancer progression.^18,19^ Hence, in addition to measuring pH changes in the cytosol and extracellular medium, it is of great relevance to precisely monitor pH variation in an endocytic organelle in cancer cells at different stages of malignancy. At present, there are several available tools to measure intracellular acidification both in the cytosol and in organelles, among which the most used are nanowire photoelectrochemical biosensors,^20−25^ microelectrodes to measure cytosolic pH, pH-sensitive fluorescent dyes that target cytosol or intracellular organelles thus allowing pH determination, and pH-sensitive proteins that can be expressed in cells reaching a specific intracellular localization where the pH is measured.^26−36^ Here, we describe an innovative method that allows precise measurement of acidification, in particular, the kinetics of acidification. Importantly, using this method, where the entry of each single sensor in endocytic organelles is recorded, it will also be possible to correlate changes of pH with other events such as membrane fusion events or else.

## Results and Discussion

### Synthesis and Characterization of Silica Microparticle-Based pH Sensors

**Figure 1:**
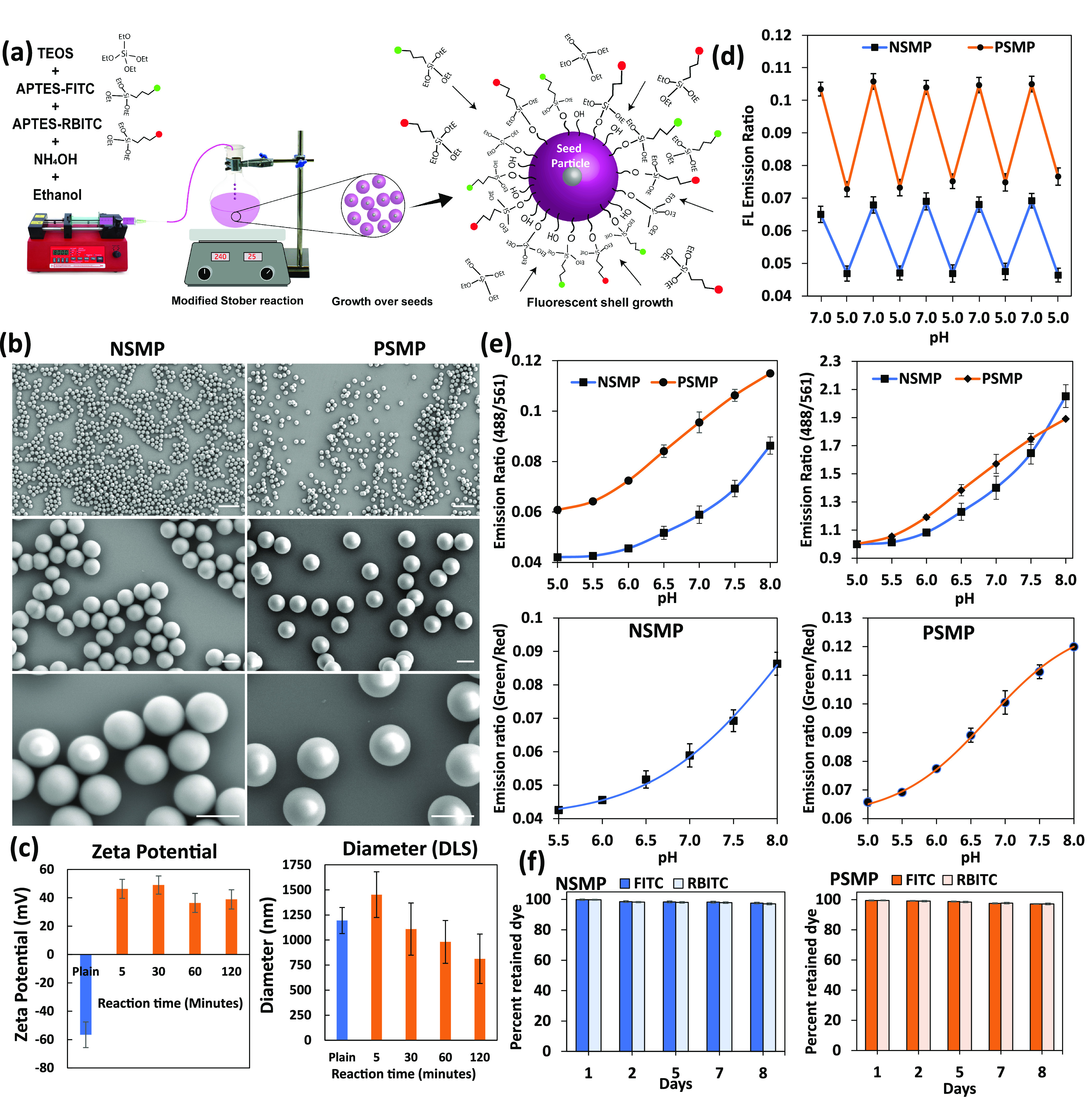
Synthesis and characterization of negatively and positively charged pH-sensitive silica microparticles. (a) Synthesis steps of pH-sensitive fluorescent silica microparticles by the modified Stöber method. (b) SEM images of (left columns) of NSMPs and (right column) PSMPs at different magnifications. Scale bars: top panels: 5 μm; middle and bottom panels: 1 μm. (c) (Lft panel) ζ Potential data showing charge values on NSMPs (plain) and PSMPs; (right panel) DLS data showing the diameter for NSMPs (plain) and PSMPs over reaction time. (d) Reversibility of fluorescence emission ratios of PSMPs and NSMPs with cyclic pH 5.0 and pH 7.0 showing the conserved sensitivity or robustness of the particles. (e) (Upper panels) Difference in fluorescence emission ratios (488nm/561nm) of PSMPs and NSMPs under known pH values. Un-normalized (left) vs normalized (right) fluorescence ratios (488nm/561nm) of PSMPs and NSMPs. (Bottom panels) Best fitting of pH vs emission ratio with Boltzmann regression for PSMPs and logistics regression for NSMPs. (f) Plots showing the retention percentage of FITC and RBITC dyes from PSMPs and NSMPs on different days of dialysis.

Silica microparticles with a capability to sense the pH ratiometrically were developed using fluorescein isothiocyanate (FITC) as the pH-sensing dye and rhodamine B isothiocyanate (RBITC) as the reference dye. The synthesis involved modified Stöber method, where initially, silica seed particles were formed followed by a slower process of growth of the particles by the slow addition of fluorescent monomers (Figure 1a). Notably, both types of synthesized particles had a spherical shape and smooth surface, in addition to being highly monodispersed and stable toward aggregation as evident from the confocal laser scanning microscopy (CLSM, Figure S1a) and scanning electron microscopy (SEM) images shown in Figure 1b. This technique of synthesis allowed tailorability over size of the microparticles, where a larger sized microparticle can be produced easily by condensation of more tetraethyl orthosilicate (TEOS) molecules on an existing smaller microparticle. An example of two different sized silica microparticles is shown in Figure S2. The diameter of the negatively charged silica microparticles (NSMPs) was estimated by fluorescence microscopy, SEM, and dynamic light scattering (DLS) analysis and was found to be 1.13 ± 0.05 μm (Figure S1), 0.93 ± 0.07 μm (Figure 1b), and 1.19 ± 0.13 μm (Figure 1c), respectively. Thus, the estimated size by different techniques results in a very similar diameter confirming the narrow size distribution. The FITC dye is known for its sensitivity toward pH change due to the presence of ionizable groups that cause a decrease in fluorescence emission as the local pH decreases.^44^ The absorbance spectrum of plain silica particles and dye-loaded silica particles is shown in Figure S1b for comparison. The nonfluorescent plain particles are known to cause light scattering, and therefore only a small part of the absorbance contributed by the dye is visible. Here, both the sensing dye and the reference dye were covalently entrapped in the silica microparticles that ensured higher stability of the sensors over prolonged applications as leaching of the dye over time can reduce the brightness and hence the sensitivity.^45^ The calculated number of FITC and RBITC molecules attached per silica microparticle are 8.92 × 10^6^ and 5.39 × 10^6^, respectively, which is equivalent to 5.77 × 10^–15^ and 4.80 × 10^–15^ g (detailed calculations are provided in the Supporting Information). In addition, silica entrapment is also known to be advantageous in protecting the dye molecules from photobleaching making the sensors more photostable.^46^ It is also noteworthy to emphasize on the size of the sensing particles as we wanted to track individual pH-sensing particles during cell uptake for intracellular pH measurements. Additionally, the size of the silica-based particles has been known to affect its cytotoxicity, where nanoparticles are found to be more cytotoxic compared to microparticles.^47,48^ Thus, we synthesized ∼1 μm sensor particles that can be easily observed and tracked using CLSM.^49^ Nanoparticle-based fluorescent sensors offer sensing capabilities^50−54^ but lack the capability of individual particle tracking due to limitations of resolution of the fluorescent microscopes. Another advantage of micron-sized silica pH sensors is their lower surface area-to-volume ratio that makes them less vulnerable toward degradation by hydrolysis compared to nanoparticles. Thus, in biological studies involving continuous tracking of the particles, all these advantages play a major role.

For intracellular pH tracking, it was essential to make sure that enough microparticles entered the cells. One effective strategy was to generate positively charged silica microparticles (PSMPs) as oppositely charged particles are known to interact more with the cells enhancing the uptake efficiency.^55^ By default, silica surface is highly negatively charged after synthesis due to the presence of hydroxyl functional groups. Therefore, repulsion between the negatively charged plasma membrane and NSMPs usually results in a poor uptake. PSMPs on the other hand tend to interact more with the cell membrane and thus get internalized to a higher extent. Here, first the negatively charged fluorescent pH sensors of silica were synthesized, and later in a one-step reaction of just 5 min, their surface was covalently functionalized with 3-aminopropyltriethoxysilane (APTES) molecules that have free primary amines. By this process, the ζ potential of the NSMPs changes from −56.6 ± 9.1 to 46.3 ± 6.7 mV (Figure 1c). During the reaction of surface modification with APTES, it was observed that the diameter of the microparticles increases by 21% in the initial 5 min and then starts decreasing significantly and get reduced by 32% compared to the diameter of bare negatively charged particles. Thus, it is important to consider the reaction duration to have positively charged microparticles without compromising the size. The effect of reaction duration on the surface charge is also important as the maximum positive charge is achieved in the initial 5 to 10 min of the reaction; after that, it gets reduced to lower values. Here, the possible reason for the reduction in size could be the dynamic equilibrium of hydrolysis and condensation reaction happening during the reaction. The surface of the existing silica microparticle is continuously hydrolyzed, and condensation of monomers from the surrounding solution on the surface of the particles happens simultaneously. At the start of the reaction, equilibrium seems to be favoring more condensation of the APTES molecules compared to hydrolysis of the surface-bound silica molecules. The same equilibrium in the later part of the reaction after 5 min may have shifted toward lower condensation and relatively higher hydrolysis causing a reduction in size. More details on the mechanism of acid-catalyzed formation of silica microparticles with a discussion on the role of hydrolysis and condensation are discussed elsewhere.^49^ Accordingly, for our intracellular pH measurement studies, we selected positively charged particles formed after 5 min of APTES coating corresponding to particles with higher positive charge (46.3 ± 6.6 mV) and ideal diameter (1.4 ± 0.2 μm).

### Role of the Microparticle’s Surface Charge on pH Sensitivity

As it is known that the ionization equilibrium of FITC or any other ionizable fluorescent molecules is highly dependent on the immediate environment, it was essential to also assess the sensitivity of the two kinds of sensors. The effect of surface charge difference on the pH sensitivity of the sensors was thus assessed by fitting the pH vs emission ratio data into sigmoidal regression functions. Both the sensors showed a good enough fit with Boltzmann function, but more specifically, NSMPs showed better fit with the logistics function (Figure 1e). This showed that there is a difference between the trend of emission ratio change and the corresponding change in pH. This became more evident from the fact that the calculated p*K*_a_ for PSMPs was 6.30 ± 0.09, while NSMPs showed a p*K*_a_ of 6.87 ± 0.15. The explanation for this could be the fact that charged functional groups on the surface of silica microparticles can interact with the FITC molecules in the vicinity by hydrogen bonding. As reported elsewhere, the entrapment of dyes in silica and the hydrogen bonding have the ability to shift the p*K*_a_.^56,57^ Therefore, FITC in this case with a p*K*_a_ of 6.5 in solution is exhibiting changed p*K*_a_ in the NSMPs and PSMPs that have a different surface charge due to the absence and presence of APTES molecules.

### pH-Dependent Reversibility of the Sensors and Stability under Cell Culture Conditions

After estimating the sensitivity of the two types of SMPs, it was also necessary to assess the reversibility of the sensors with fluctuating pH values. Therefore, we monitored the fluorescence emission ratios for both the sensors in L15 medium where pH was fluctuated between pH 7.0 and pH 5.0, and samples were collected for each pH value. This extreme variation in pH in a cyclic fashion is a very good test for checking the robustness of the sensors as poor sensors will show significant variation and will show different readouts for the same pH value after few cycles of pH fluctuations. Here, with both NSMPs and PSMPs, the cyclic pH variation resulted in appreciably repeatable fluorescence emission ratios against corresponding pH values (Figure 1d). This proved that both the sensors are very stable and can detect pH variations under highly dynamic conditions such as cellular microenvironment and endocytic journey which involve continuous variation in pH. Here, it is interesting to note that the percent change in fluorescence emission for both sensor types is very similar for the pH variation. This shows that the overall sensitivity is conserved in both sensors. As the cellular studies involved time lapse microscopy experiments in aqueous solution, it was essential to assess the stability of the pH microsensors in terms of leaching of the two dyes over time. Therefore, we performed the dialysis-based dye release study and found that compared to the amount of dye loaded in the microparticles, only ∼3% dye molecules were released in deionized water over the period of 8 days for both NSMPs and PSMPs. Here, the micrometer size of the particles could be the possible reason for slower degradation of the surface as the surface area/volume ratio is considerably lower for microparticles compared to nanoparticles (Figure 1f). Although silica-based particles are considered inert due to their nontoxic degradation byproducts, there could still be cytotoxicity due to their interaction with cellular proteins. Thus, we assessed the cytotoxicity for both NSMPs and PSMPs using three different cell lines, namely, L3.6pl,MIA PaCa-2, and PSE cells for 24 h using the MTT assay. The results are shown in Figure S3 where both NSMPs and PSMPs resulted in more than 80% viability for all three cell types even at the highest concentration of 500 particles per cell.^58^ This proves that both types of microsensors are cytocompatible and could be used flexibly even at desired higher concentrations.

### Application of PSMPs for Intracellular pH Sensing

**Figure 2:**
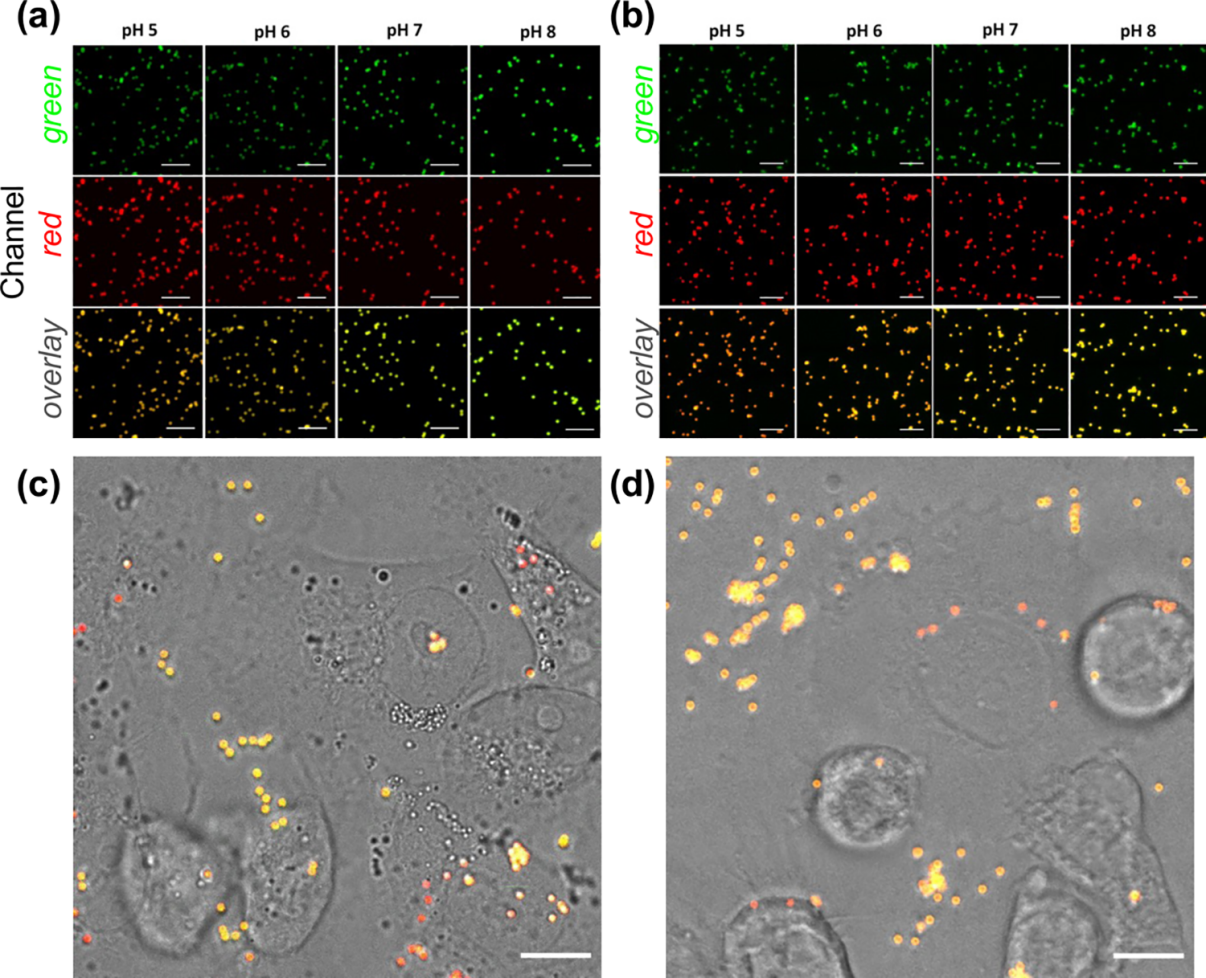
Comparative change in the emission of NSMPs and PSMPs at different pH values. In vitro pH response of PSMPs following cellular uptake. (a,b) CLSM micrographs showing the pH dependence of (a) NSMPs and (b) PSMPs fluorescence in pH-adjusted cell medium (FITC was excited at 488 nm and RBITC at 543 nm). Green channel (Em: 500–550 nm), red channel (Em: 570–700 nm), and overlay of the two fluorescence channels are reported. (c,d) Fluorescence micrographs showing the color changes of PSMP sensors added to (c) MDA-MB-231 cells and (d) MCF-7 cells, as recorded after 24 h (ratio PSMPs/cells = 8:1). Before internalization, extracellular PSMPs display a strong yellowish fluorescence due to the neutral pH of the cell medium. After internalization, the PSMP sensors display a strong red fluorescence due to their confinement in acidic endosomal/lysosomal compartments inside cells. Images were taken in green and red fluorescence and transmission channels. The overlay of the three channels is presented in (c,d). Scale bars in (a–d): 10 μm.

**Figure 3:**
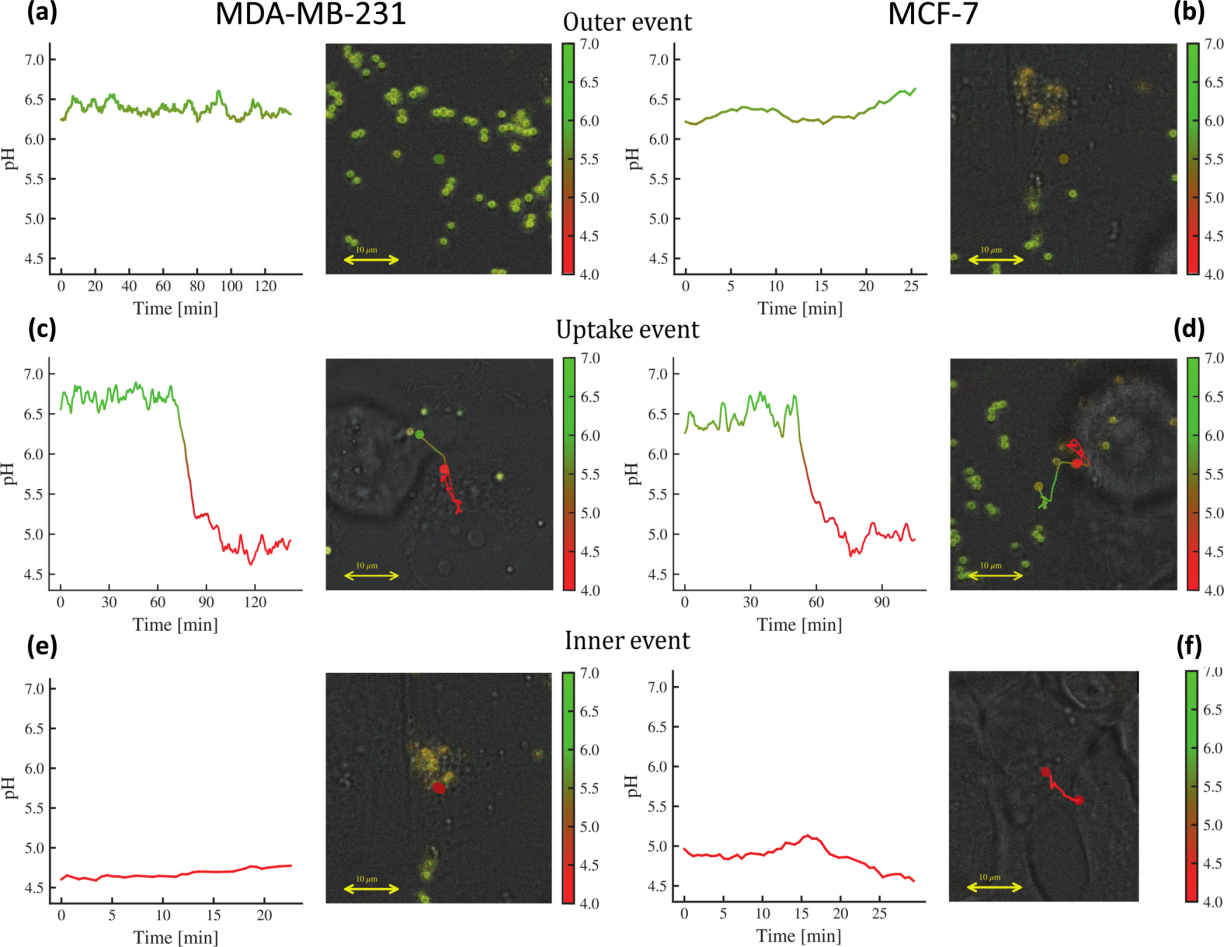
Examples of the three prototypical recorded events. (a,b) Lack of caging (outer) as the probe remains confined outside the cell. (c,d) Caging event as the probe is captured by the cell. (e,f) Lack of caging (inner) as the probe is already inside the cell.

The PSMPs were selected for all intracellular pH tracking analyses due to their net positive surface charge and higher efficiency of uptake. Another advantage of PSMPs is their slightly lower p*K*_a_, compared to NSMPS, that makes them better candidates for sensing acidic pH in the endosomes and lysosomes over different internalization stages. The main objective is to track the pH changes over time while the microparticles are entering the cells and getting confined within the endosomes (pH 6.8–4.9)^59^ and then in lysosomes characterized by pH 4.5–5.0.^33^ Before incubating the PSMPs with living cells for microscopy analyses, it was necessary to prepare a calibration curve using fluorescence images of PSMPs at predetermined pH values. The data derived from the fluorescent images were used to generate a ratiometric image using the lab-built algorithm which resulted in ratio values for each pH. The fluorescence microscopy images for NSMPs and PSMPs are shown in Figure 2, panel a and panel b, respectively, where the merged images clearly show the change in color of the microsensors corresponding to change in pH values. We selected MCF-7 and MDA-MB-231 cell lines for our study of monitoring the rate of endosomal and lysosomal pH changes during the uptake process to see if we can correlate the difference in their aggressiveness with the rate of endosomal acidification. This is considered important as endosome and lysosome pH are directly related to the activity of hydrolytic enzymes, autophagy, and survival in the highly acidic environment of tumors, aiding the development of drug resistance, as well as crucial roles in tumor cell invasion, migration, and metastasis.^62,63^ The strategy used was to first assess the confinement of PSMP sensors into acidic compartments of both MCF-7 and MDA-MB-231 cells (Figure S4) and second to incubate the PSMPs with the two cell lines independently and record the particle fluorescence intensities using time lapse fluorescence microscopy over time. Continuous monitoring resulted in three types of events (see Figure 3): the first type of event is the case where the particles remain outside the cell throughout the experiment (thus, their fluorescence emission remains unperturbed, fluctuating around a typical value); the second type of event consists of PSMPs that are already inside the cells since the starting of recording (hence, they also store a typical lower pH value which fluctuates for the whole time of image acquisition). Finally, the third and most interesting situation consists in particles that show a sensible variation in the fluorescence emission intensity (coupled with a similar variation in their location over time): an uptake event whose statistics we aim to collect. Should this be the case, the PSMPs appeared greenish yellow in the start of their journey toward the cells, became orange while crossing up to red once inside the cell where the recorded pH, likely of the endosome, reached its minimum as a result of quenching of FITC and insensitivity of RBITC under acidic pH (Figure 2c,d).

### Collecting the Caging Events via Automatic Classification

Given the large amount of recorded and detected tracks, an entirely automatic classification of the outcome events is a mandatory request: as stated, we expect to face (and correspondingly classify) three prototypical situations:

- Outer event (OE) The probe stays outside of a cell for the whole observational time and it reads the pH in the cell surroundings, but it does not get captured by the cell (Figure 3a,b).
- Caging event (CE): The probe initially stays outside the cell and measures the pH in its surrounding; then the cell engulfs the probe and the latter reads the variation of the pH from outside the cell to inside the cell: this is the key event whose statistics we aim to collect (Figure 3c,d).
- Inner event (IE): The probe stays inside a cell for the whole observational time, and it reads the pH inside the cell and stays confined within the cell (Figure 3e,f).

**Figure 4:**
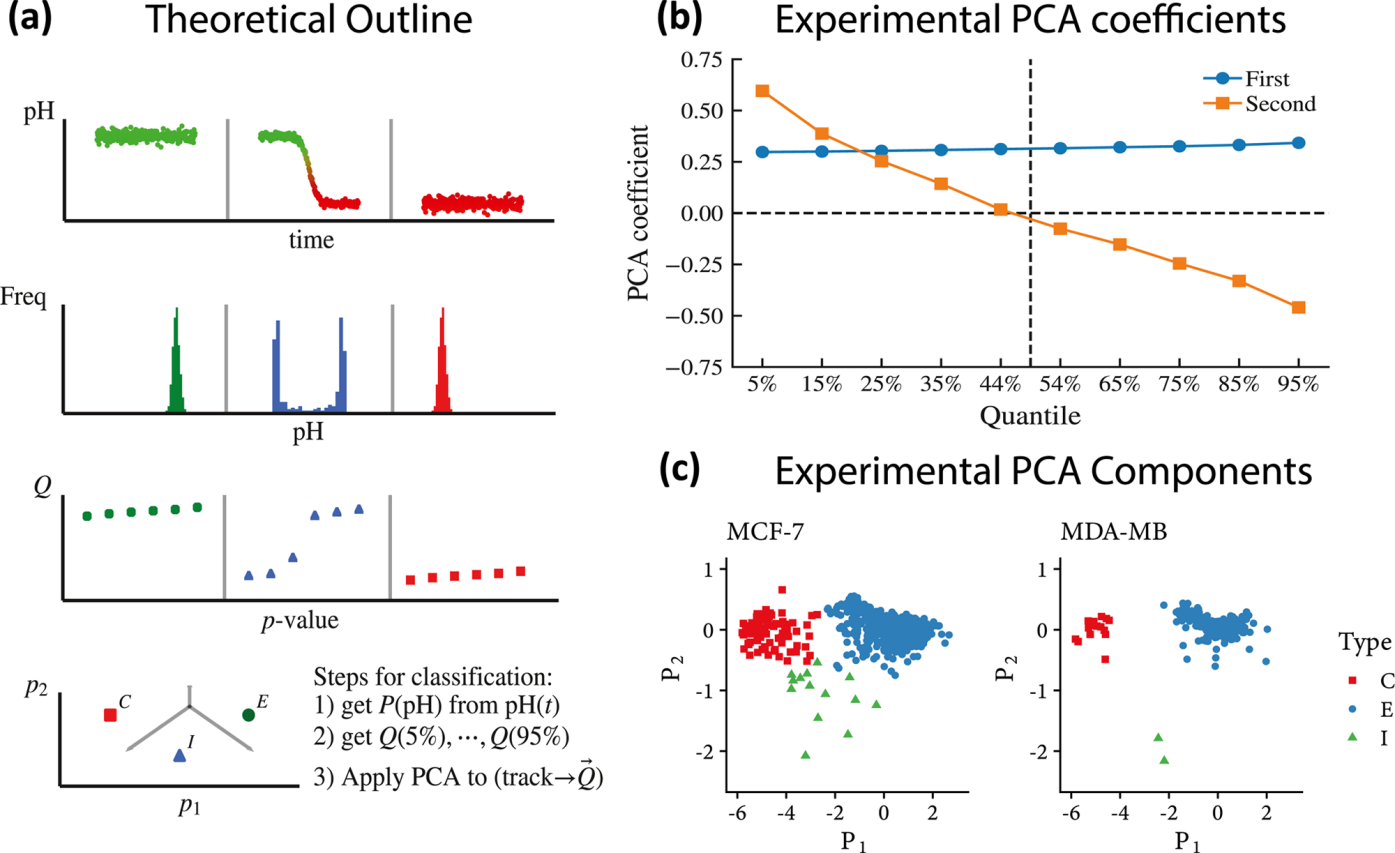
Automatic classification of the caging events. (a) Moving from upper plots to lower ones, in the top row, three archetypal evolutions of pH vs time are shown (mimicking, respectively, an outer, an uptake, and an inner event). In the successive line, this dynamical information is converted into static histograms: crucially, while caging is coupled with a bimodal distribution, outer and inner events (obviously) result in monomodal distribution, and this observation is the core of the automatic classification. In the third row, we dissect the obtained distributions in terms of 10 quartiles: this allows for PCA over the latter. Finally, in the bottom row, we have a phase diagram where this classification naturally shines in the plane of the first two principal components. (b) Decomposition of the two principal components in terms of the original quantiles. (c) Cluster’s emergence (by automatic detection) of the various events on the datasets reported for both the cellular lines.

Obtaining this kind of automatic classification unfortunately is not standard in the computational literature, and hence we had to build the algorithmic implementation from scratch for the following idea: automatic classification, that is, *cluster detection*, best work with images^65^ and hence we aim to collapse all information stored in the datasets on a plane. Despite being tricky, this is possible and results in the following procedure to be applied to any track (namely, for any reading probe, on the whole time-ordered vector of its recorded pH values):

1 We take the probe’s output vector (i.e., the function pH(*t*), where *t* stands for time) and we calculate the histograms of these recordings, discarding their temporal order but, rather, looking solely at the measured pH values: see the first two rows of panel a in Figure 4. Crucially, while IE and OE are coupled to monomodal distributions, CE is related to a bimodal distribution.
2 We calculate the quantiles (*Q*) of these empirical distributions at regular intervals, namely, via 10 values uniformly spaced in the interval [5%, 95%] (these can easily be shown to satisfactorily characterize any nonpathological distribution): in the third line of panel a in Figure 4 each pH track is characterized by 10 coordinates, namely, the 10 values of the populously of their respective quantiles. Note that at this point, we compressed the complex dynamics of a recorded event as a point in a 10-dimensional space.
3 Finally, to further reduce the above dimensionality, we apply principal component analysis (PCA) to collapse the number of effective coordinates up to just two (the first two principal components, keeping overall ∼90% of the initial information), and with planar plots, we finally achieved a cluster representation of the possible outcomes that can be inspected even by trivial visual check. Note that, to infer the clusters in the planar projection of the first two PCA eigenvectors, we used the unsupervised protocol K-means++.^64^ See the last line of panel a in Figure 4, while the PCA coefficients for the two most important components are depicted in Figure 4b and an example of the clusters for both the cellular lines is depicted in Figure 4c. Note that the PCA is not at work in the space of the trajectories, rather in the space of the vectors storing the quantiles: PCA takes as input these 10-entry vectors, one per probe, and diagonalizes them such that the first eigenvalue (the first PCA) roughly carries information about the average pH read along the whole trajectory stored by the probe, while the second roughly returns a measure of dispersion around that value, and it is the interplay between these two numbers to provide enough information for inferring a CE, as long as the distributions are monomodal (hence no caging events) and pH mean values are at extreme values and the relative dispersion is low; when there has been a CE, the distribution of the reads become bimodal, the mean value lies in between the two peaks of the distribution, and the dispersion is huge.

**Figure 5:**
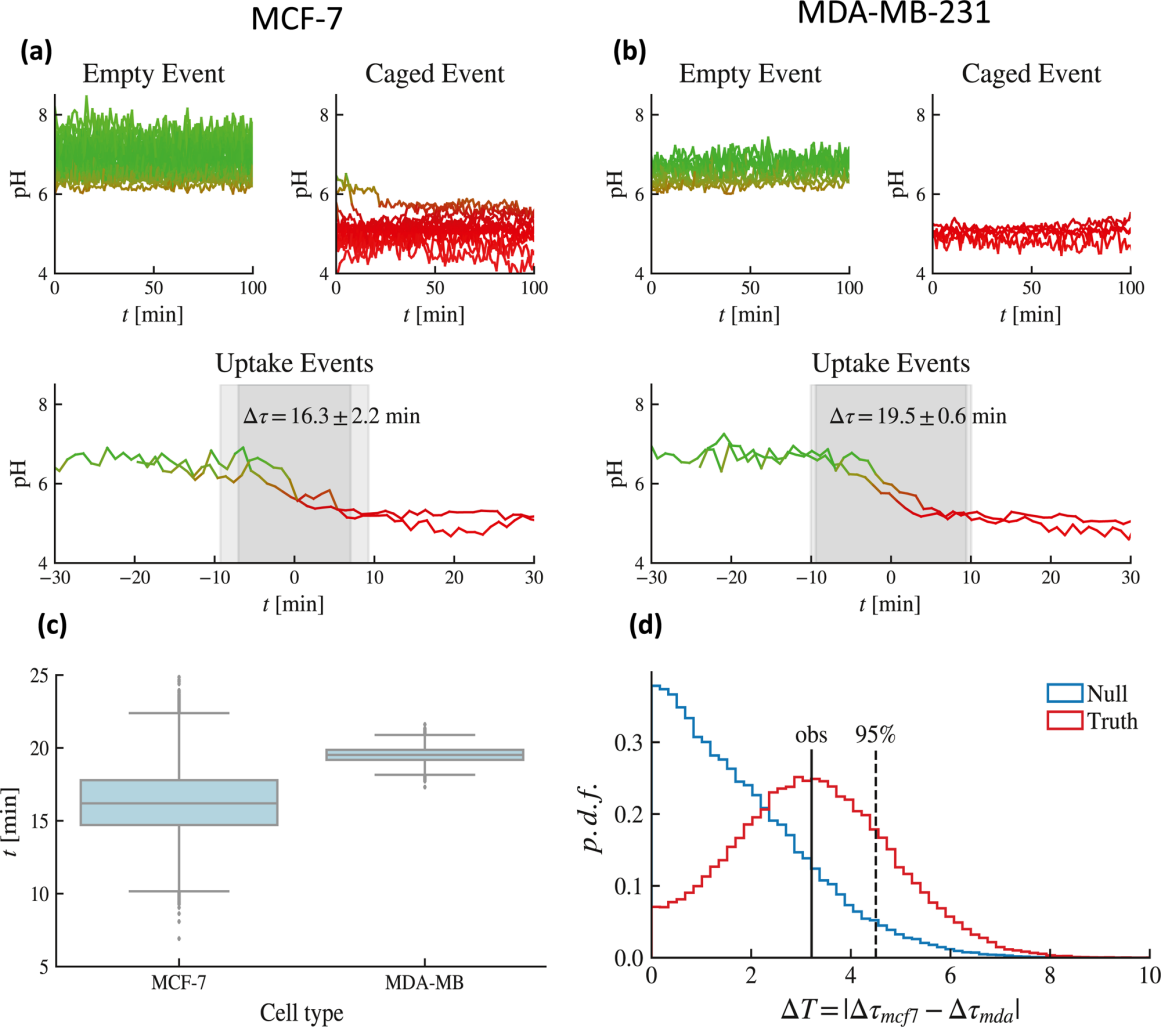
Estimates of the acidification time. (a) Outer, inner (top), and caging (bottom) evolution of recorded pH vs time for MCF-7 cells. (b) Outer, inner (top), and caging (bottom) evolution of recorded pH vs time for MDA-MB-231 cells. (c) Box plot of the distribution of the acidification time: while the mean are somehow close, resulting in Δτ = 16.3 min for the MCF-7 type and Δτ = 19.5 min for the MDA-MB-231 type, the former exhibits a much broader variability as the relative standard deviations read as 2.2 min vs 0.6 min. (d) Hypothesis test on the statistical value of the difference between the two acidification times suggesting that the temporal gap between the two average acidification times related to the two cellular types is not significant.

The method described has been successfully applied to datasets acquired for both the cell types, namely, MDA-MB-231 cells and MCF-7 cells; in Figure 5 panel c, we can observe that both datasets cluster nicely, thanks to the strategy described above: this automatic classification allowed to move directly to the analysis and characterization of specific event types such that, in the next section, we can extract a robust estimate of the acidification time for both the cellular types.

### Estimates of Acidification Time

For estimating the acidification time of these two cell types, now we focus only on the uptake events. As a result of improved probes, readings are more specific, but they also fluctuate more than in previous cases; hence, rather than the standard sigmoidal fit (e.g., in ref (67) they used ), to infer the mean acidification time, we rely on the isotonic regression (IR),^67^ whose job is to find the nonincreasing curve that best models the input data, and we apply it to each uptake track. Introducing λ as a free parameter for the fit, for each curve pH_iso_(*t*), we search for solutions in time of the form
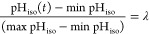
2.1where 1 – λ represents the percentage of pH drop, effectively obtaining a function *t*(λ) that maps a pH drop to a particular time. Finally, we define the acidification time Δτ as standard, namely
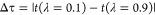
2.2Within this setting, estimates of the acidification time—as sketched in Figure 5a,b for both the cell types—have been obtained; see Figure 5c,d. A direct comparison of the results clearly shows that the mutated cell type MCF-7 spans a much broader range with respect to the MDA-MB-231 cell type.

## Conclusions

This work describes a very effective and accurate methodology to monitor acidification kinetics of endosomes in cancer cells. The endocytosis and fluorescence emission of individual micrometer-sized pH-sensitive particles were monitored using time-lapse CLSM. The studies were conducted using PSMPs owing to their higher uptake probability to observe more uptake events. The particles were synthesized using the modified Stöber process and were later processed to make their surface positively charged. Owing to dual emission by covalently entrapped FITC and RBITC, the sensors are ratiometrically pH-sensitive and thus are more accurate. The micrometer size makes it possible to track individual particles and also stabilizes them against degradation. The particles were characterized using different techniques for their size, shape, pH sensitivity, charge, long-term stability, and cytocompatibility and were found to be very suitable for current and similar studies where the endocytic acidification dynamics needs to be studied.

This novel generation of high-performing probes required novel related computational approaches able to efficiently deal with the massive datasets that were generated by time-lapse CLSM . At first, an algorithm for particle detection and tracking has been adapted starting from the reference provided in ref:^37^ as in the original paper, the authors also consider cellular division (that is not observed on the timescale we analyze), and we simplified their algorithm by removing that part. This resulted in an improved version for the present case, further tracking inference resulted essentially robust w.r.t. the size of the particle to reveal and track, allowing for more versatility at work with heterogeneous probe structures. We tested this algorithm (as extensively discussed in the Methods B section) at work on synthetic datasets generated by the Vicsek model (that is a standard reference model for self-propelled particles used to model active matter^38^) at various densities obtaining a percentage of error lower than 10^–5^ with *O*(10^2^) particles. Further, this percentage always scales polynomial in the system size (linearly or parabolically, depending on the density, as discussed in the Supporting Information), but in any case, always extremely fast if compared with NP-algorithms. Beyond refined algorithms for particle detection and tracking (that are almost independent of the size of the particle to reveal and track, allowing for more versatility at work with heterogeneous probe structures), the core novelty at the computational level lies in an automated algorithm for cataloging outcomes of the possible interactions cell probe: these result in three archetypical scenarios, namely, *outer event* (the probe is always confined outside the cell), *inner event* (the probe is always confined inside the cell), and *uptake event* (the probe is captured by the cell). As the last case is the most important, yet the most rare, prescreening via automatic classification of events (in order to retain just uptake events for further analysis) was mandatory and resulted in an extremely fast and cheap algorithmic protocol: for each probe’s trajectory, we have a time-ordered vector of pH readings whose temporal organization we completely disregard and rather we focus at the distribution of these readings. If the latter is monomodal, the probe under study is entirely confined outside or inside the cell (center, respectively, at higher or lower pH mean values); if the distribution turns out to be bimodal that is the hallmark of the caging event, this observation, coupled to PCA compression, is pivotal for automated event selection.

Finally, for these uptake events, via IR algorithm, we best-fitted the acidification time for the two cellular lineages, while the mean acidification time is relatively comparable among MCF-7 and MDA-MB-231 cells, the variance in the former is by far larger than the variance in the latter, revealing a highly heterogeneous behavior of the MCF-7 cellular line if compared with the MDA-MB-231 counterpart. The heterogeneity observed between MCF7 and MDA-MB2-31 could be related to the different types of tumors from which these cells derive. Indeed, other works showed a greater heterogeneity for the expression of several markers in luminal-like tumors compared to more aggressive ones and MCF7 derived from luminal-like tumors.^39−41^

The successful application of particle-based optical sensors combined with our computational approach provides a new and rapid route to precisely quantify intracellular acidification in different cancer cells.

## Methods A: Experimental Protocols

The experimental methods used to generate the dataset can be summarized by the following series of building blocks discussed in this section:

- Description of probe fabrication
- Culturing cells for microscopy and microparticle addition
- Fluorescence microscopy

### Description of Probe Fabrication

#### Synthesis of pH-Sensitive NSMPs

Dye molecules FITC and RBITC were covalently linked with APTES in ethanolic solution for 3 h in dark. Briefly, 5 mg of FITC and 6.5 mg of RBITC were dissolved separately in 3 mL of anhydrous ethanol followed by addition of 13 μL of APTES. The two solutions of respective dyes with APTES were kept on magnetic stirrers for 4 h at room temperature. The formed FITC–APTES and RBITC–APTES conjugates were used directly in the next step without further purification.

The silica microparticle formation starts with the formation of silica seed suspension (step 1) followed by its growth (step 2) by slow addition of the monomer TEOS and dye-APTES conjugated molecules (Figure 1). Briefly, the seed formation was initiated by dissolving 23 mg of KCl in 9.45 mL of deionized water in a round-bottom flask followed by addition of 96 mL of absolute ethanol, 6 mL of ammonium hydroxide (28%), and 1.73 mL of TEOS. The solution in the flask was stirred using a magnetic bead at 240 rpm for 30 min. The formation of seed particles over this time can be seen by observing the transition of the transparent solution, which becomes extremely turbid with white color. The next step involved increasing the size of the seed particles by slow addition of monomer TEOS along with dye-conjugated APTES. Here, 4.4 mL of TEOS, 2 mL of FITC–APTES, and 2 mL of RBITC–APTES were dissolved in 33 mL of absolute ethanol. The equimolar concentration of both FITC and RBITC dye molecules was used in the reaction mixture. The thought of using equimolar FITC and RBITC is supported by previous report^42,43^ where a higher ratiometric pH sensitivity is observed with molar feed ratios close to 1:1, whereas a decrease in pH sensitivity was observed with an increase in the number of RBITC molecules compared to FITC. Therefore, we finalized the best combination of 1:1 M ratio for synthesizing our sensors.

This solution was then slowly added drop by drop (flow rate: 0.05 mL min^44^) into the seed solution that was stirred at 240 rpm using a 50 mL plastic syringe. During the seed formation and growth of the fluorescent silica particles, the flask was airtight to prevent diffusion of ammonia. The reaction proceeded for 24 h followed by carefully pipetting out the supernatant leaving larger debris of aggregated particles in the bottom. The collected particles in the supernatant were washed three times in ethanol using centrifugation at 2000 rpm followed by washing with deionized water thrice. The washed particles were finally stored in ethanolic suspension at 4 °C.

Fluorescent silica microparticles with controlled size can be made simply by diluting the seed particles that are synthesized in the first phase of the Stöber reaction. Afterward, the slow addition of the monomers cause growth on the seeds resulting in microparticles with a larger diameter. In this way, the size can be increased with a decrease in the total number of particles. The effect of size on the sensitivity of the microparticles toward pH change is also a matter of consideration. Briefly, to synthesize ∼2 μm silica microparticles, only 25% volume of the seed dispersion is taken from step 1 for starting step 2 of the particle synthesis that consist of growth of the particle. This causes an increase in the final size of the particles as there are relatively less growth centers causing availability of more monomer per seed particle. The remaining steps to be followed are the same as previously mentioned. To further increase the size, the particles formed of a certain size can be used as the seed for the next reaction, and therefore the growth due to more addition of monomer causes further growth of the pre-existing particle.

#### Synthesis of pH-Sensitive PSMPs

The synthesis of PSMPs was achieved by the development of an additional layer of APTES onto the negatively charged surface of NSMPs. The APTES-coated NSMPs attain a positive charge due to the presence of primary amines of the APTES molecule. Briefly, 2 mL of APTES and 200 mg of FITC–RBITC NSMPs were dispersed in 40 mL of deionized water containing 2.5 μL of glacial acetic acid. The reaction was kept on stirring, and the samples were recovered after 5, 30, 60, and 120 min followed by washing of microparticles in ethanol and water three times each. The washed particles were finally stored in ethanolic suspension at 4 °C for further use.

#### Characterization of the SMPs: Estimation of Size

(a) Light microscopy: The size of the microparticles was estimated by using light microscopy including bright-field and fluorescence microscopies. The silica microparticles were dispersed in deionized water and imaged using 8-well IBIDI chamber slides with lid (cat. no.: 80826). Bright-field microscopy was performed using an Invitrogen EVOS digital inverted brightfield and phase contrast microscope. The fluorescence microscopy for size estimation was done using a Leica SP8 confocal microscope. The image acquired by microscopy was analyzed using ImageJ to calculate the diameter, where thresholding was done followed by particle size analysis to get the area of individual particles. Finally, the mean diameter was calculated from the area with standard deviation.
(b) SEM: Sample preparation for SEM involved dropcasting 10 μL of the SMP dispersion in ethanol on silicon wafers cleaned by deionized water and ethanol followed by overnight drying at room temperature. The samples were analyzed directly without gold sputtering. The SEM imaging was performed by the MERLIN Zeiss SEM instrument at an accelerating voltage of 5 kV using a secondary electron detector (SE2).
(c) DLS: The size estimation by DLS involved making a dilute dispersion of microparticles in deionized water. Analysis was done in a 3.5 mL plastic cuvette using a Zetasizer Nano ZS90 (Malvern, USA) equipped with a 4.0 mW He–Ne laser (633 nm) and with an avalanche photodiode detector. Deionized water was used as the dispersant (*n* = 1.33, η = 0.88), and measurements (number of measurements 20, number of cycles 3) were performed at 25 °C. The refractive index of the SMP used during the acquisition was taken as 1.5.

#### Characterization of the SMPs: Estimation of Charge

Charge on hydroxyl-coated silica (NSMPs) and primary amine-modified silica microparticles (PSMPs) was assessed using a Zetasizer Nano ZS90 (Malvern, USA) equipped with a 4.0 mW helium–neon laser (633 nm) and with an avalanche photodiode detector. Folded Capillary ζ Cell was used for loading the sample. Deionized water was used as the dispersant (*n* = 1.33, η = 0.88), and measurements were performed at 25 °C. The refractive index of SMPs used during the acquisition was taken as 1.5.

#### Characterization of the SMPs: Estimation of Fluorescent Properties

The fluorescence spectra of SMPs dispersed in Leibovitz’s L-15 Medium (without phenol red) was recorded using a spectrofluorimeter (Cary Eclipse). The pH of the dispersion was adjusted to 5.5 and 8.0 values by using 1 N HCl and 1 N NaOH. The samples were excited at 488 and 561 nm corresponding to FITC (emission 505–550 nm) and RBITC (emission 575–700 nm), respectively (Figure S1c,d). The excitation and emission slit widths of 5 nm was used for all measurements. The emission ratio under different pH values was calculated by taking the ratio of green and red fluorescence intensities.

#### Fluorescence Reversibility under Cyclic pH

The robustness of the SMPs was assessed by analyzing their fluorescence emission ratio under two different pH values (pH 5.0 and pH 7.0) in a cyclic fashion. The readings were recorded using a spectrofluorimeter (Cary Eclipse). The pH of the SMPs containing dispersion in L15 media was varied by using 1 N NaOH and HCL. Sample was collected at pH 7.0 and pH 5.0 in a cyclic fashion and analyzed using the fluorescence spectrophotometer.

### Culturing Cells for Microscopy and Microparticle Addition

MCF-7 and MDA-MB-231 are ATCC cell lines grown in Dulbecco’s modified Eagle’s medium (DMEM) supplemented with 10% fetal bovine serum (FBS), 2 mM l-glutamine, 100 U/mL penicillin, and 10 mg/mL streptomycin in a 5% CO_2_ incubator at 37 °C. Cell lines were routinely checked for mycoplasma and confirmed mycoplasma-free. The reagents for tissue culture were from Sigma-Aldrich (St-Louis, MO, USA) or Gibco (Gibco, Grand Island, NY, USA).

2.5 × 10^4^ cells were plated per well in 8-well IBIDI chamber slides (plastic bottom) in DMEM media supplemented with 10% FBS for overnight culture in an incubator maintained at 37 °C under 5% CO_2_. Before imaging, the DMEM medium was replaced with L15 medium containing 2.0 × 10^4^ PSMPs. The media containing the SMPs was prepared by vigorously vortexing PSMPs in L15 media for 1 min. This helped in separating particles that were loosely adhered with each other. As the particles settle to the bottom over time, the vial containing PSMPs was vortexed gently before addition to the cell culture chamber slides to maintain a homogeneous dispersion of SMPs.

### Immunofluorescence and CLSM

MCF-7 and MDA-MB-231 cells were grown on coverslips and exposed to PSMPs, as described in Section 4.2, for indicated times. Then, the samples were processed for immunofluorescence as described previously.^60^ In brief, the cells were fixed with 4% paraformaldehyde and permeabilised with 0.2% saponin. The samples were then incubated with anti-LAMP1 antibody (Abcam, ab24170) at 4 °C overnight followed by second antibody Alexa Fluor 647 (Invitrogen, A-31573). The coverslips were then mounted in the mounting media (16% [wt/vol] Mowiol 4–24 [EMD Millipore] and 30% [vol/vol] glycerol in PBS) and analyzed under a confocal laser scanning microscope (LSM700; Carl Zeiss; 63× oil-immersion objective (1.4 NA)) (Figure S4). Images were processed using ImageJ software. The line scan analysis was performed as described previously^61^ and adopted for the indicated experiment. Specifically, a line was drawn in the middle of the LAMP1 positive structure and the PSMPs. Then, the fluorescence intensity of each stained object along this line was plotted. Excel was used for data analyses and graphing. Adobe Photoshop CS3 was used to adjust the contrast of the images (for presentation only), whereas Adobe Illustrator CC 2014 (Adobe Systems) was used to illustrate figures.

### Live-Cell CLSM

Fluorescence CLSM was performed using a Leica SP8 microscope. The lasers used for excitation were laser line 488 nm for exciting FITC and laser line 561 nm for excitation of RBITC. The pinhole used for the imaging was 2.51 airy unit corresponding to a section thickness of 2 μm. The emission was collected using a 63× objective (HC PL APO CS2 63×/1.40 OIL) ,and the detector was Hyd sensor (Hyd2, 500–550 nm) for FITC and PMT (PMT3, 470–700 nm) for RBITC.

The read mode was in-line using the resonant scanner (8000 Hz) to reduce photobleaching and to minimize between frames movement. A zoom of 5× was used, and tiling was done to create a 5 × 5 matrix, where the first tile was used to perform autofocus for each time point. The 5 × 5 matrix was autostitched by the Leica LAS AF software. As the cells have a height of few microns, there was a chance of losing the particles from focus when they were entering the cells.

Therefore, to avoid losing events, *z*-stack was acquired using a resonant scanner (scanning rate 8 kHz). This helped reducing the gap between the time points of consecutive images. A faster imaging helped in accurate tracking of the particles due to reduced tracking errors because of less untraceable particle movements. The time-lapse fluorescence confocal imaging was performed every 30 s for 1–3 h to observe events of particles uptake by the cells and subsequent change in their fluorescence emission. The imaging experiments were performed at 37 °C, and the incubator was set to this temperature 1 day before the acquisition to reduce the focal drift caused by thermal expansion of the microscope optics.

## Method B: Computational Protocols

The computational methods used to analyze the dataset can be summarized by the following series of building blocks discussed in this section:

- Calibration of sensors
- Particle detection
- Ratio extraction
- Particle tracking
- Track classification
- Track characterization
- Validation

### Calibration of Sensors

**Figure 6:**
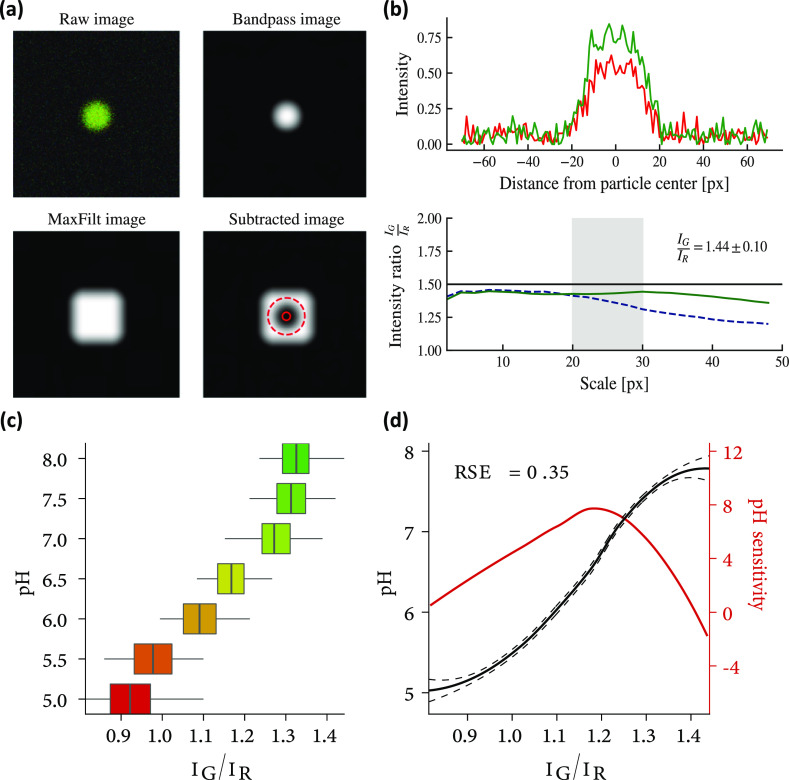
Converting a light signal into a chemical measurement. (a) Summary of the four main steps to identify a sensor. The original image (top left) is filtered by a low-band filter (top right) to detect the maximum intensity pixel, locating the probe. This white sphere is then enlarged by a max-filter algorithm (bottom left), and by subtracting the original image from the resulting white square, the final image (bottom right) is obtained. (b) Intensity of the original image as presented in the previous (top) panel. Different methods to evaluate the ratio *I*_G_/*I*_R_: the ideal case is the black horizontal line, a standard algorithm (e.g., the direct evaluation of the ratio between the two intensities) behaves as the dotted blue line, and the green line traces the behavior of our algorithm, over-performing with respect to the standard route.^66^ Note that the gray area ranges from 20 to 30 pixels as, by a glance at the upper plot, it is evident that the probe is occupying at least 20 pixels and reasonably no more than 30. (c) Check that the new approach to probe identification and light measuring produced a monotonic calibration curve. (d) Refined interpolation of the calibration curve shown in panel (c) equipped with error bars at the confidence level of ±1σ (continuous black line and relative dotted black lines) and its sensibility curve (i.e., the derivative of the pH vs the ratio of intensities) aiming to highlight where (i.e., at which values of *I*_G_/*I*_R_ ratios) the probes are best performing.

Constructing a sound calibration curve is a mandatory prerequisite to calibrate the ratiometric sensors, and seven gels at different pH (5 → 8 with steps 1/2) were prepared equipped with our sensors; these gels were imaged using a confocal microscope, and particle detection and ratio quantification were performed via the methods described in the following subsection. The results are depicted in Figure 6 panels c and d [the smooth curve in the latter is the result of locally estimated scatter-plots smoothing local regression^a^ applied on the calibration dataset made up by the pairs (ratio *I*_G_/*I*_R_, nominal pH of the gel)]: note that the calibration curve is a monotonic invertible map that allows to link one-to-one ratios of readings (of luminescence intensities) to pH values. Note further that, given its sigmoidal shape, solely away from the saturation regimes (i.e., far from pH values lower that pH_min_ ∼ 5.2 and higher than pH_max_ ∼ 7.7), we obtain a segment of pH conversion 5.2 ≤ pH ≤ 7.7 where there is a quasi-linear relation bridging intensity measurements and pH values: all biological dynamical processes we focus on in this manuscript take place in that delta.

### Particle Detection

The algorithm for detecting particles and calculating their positions is based on a simple three-step process.

1 Take the raw, original, image and transform it in grayscale (i.e., black and white, BW) by summing the intensities in each channel. In order to filter out unwanted noise, apply a band-pass filter on the BW image just produced: the particular form of spatial band-pass filter we use is realized by first convolving the grayscale image with a Gaussian kernel, that is

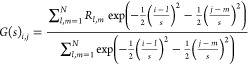
5.1where *s* is the spatial scale of the filter, *R*_*l*,*m*_ is the intensity of the grayscale image at pixel position *l*, *m*, and *G*(*s*)_*i*,*j*_ is the intensity of the Gaussian blurred image at pixel position *i*, *j*. Afterward, we convolve the grayscale image with a boxcar filter realized by
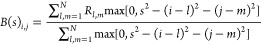
5.2where *s* is the spatial scale of the filter and *B*(*s*)_*i*,*j*_ is the intensity of the Gaussian blurred image at pixel position *i*, *j*. The final band-passed image is realized by subtracting *G* and *B* in the following way
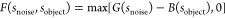
5.3where *s*_noise_ and *s*_object_ are, respectively, the characteristic scales of the noise in the original image and the scale of the particles to be detected.

2 On the band-passed image *F*(*s*_noise_, *s*_object_) (which we call simply *F* from now on), we apply a local maximum filter of size 3 by 3 pixels which is realized in the following way

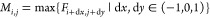
5.4

3 By comparing *F* and *M*, we can detect the positions of the particles by choosing only the positions *i*, *j* where *F*_*i*,*j*_ = *M*_*i*,*j*_ and *M*_*i*,*j*_ > λ × max{*F*_*ij*_|1 < *i*, *j* < *N*} where λ is a relative threshold (0 < λ < 1) for selecting the local maxima (e.g., particles).

The whole procedure is illustrated in Figure 6a where we show the result of the three steps individually for a random example.

In order for the method to properly work, one must tune *s*_object_, *s*_noise_, λ with some care. After having correctly identified the position of each particle inside the image, the next step is to calculate its intensity ratio starting from the intensity values attained in the unfiltered original image in the red and green channels: the procedure for doing so in a reliable way is depicted in the next subsection.

### Ratio Extraction

To extract properly and automatically the ratiometric measurements of the channel intensities, we elaborated an algorithm based on the minimization of their geometric ratio: for each channel *C* = (*G*, *R*), we perform a weighted average of the logarithm of the intensity of that channel *I*_C_, weighted by *I*_C_ itself operatively resulting in the formula
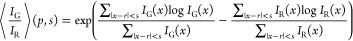
5.5where *s* is the spatial scale of the particle, *p* = (*p*_*i*_, *p*_*j*_) is the pixel position of the center of the particle, and *I*_R(G)_(*x*) is the intensity in the red (green) channel at pixel position *x* = (*x*_*i*_, *x*_*j*_). In older literature,^66^ the method for calculating the ratio was based on the formula
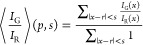
5.6this method was proven unreliable in the case of silica particles since their size distribution is broader and the simpler method is more affected by the choice of the scale.

To confirm the improved resolution of our algorithm quantitatively, we generated a synthetic example with a known true ratio *I*_G_/*I*_R_ ≔ *R*_true_ between the channels fixed at *R*_true_ = 1.5, and we show in the panel b of Figure 6 that our method properly recovers the true signal (resulting in an inferred value *R*_infer_ ∼ 1.44 ± 0.10) and the read is roughly invariant with respect to a particular choice of scale (i.e., the curve *I*_G_/*I*_R_ vs pixels is almost a constant): the new approach is almost unaffected by the variance in the sensor size (and the systematic bias affecting both methods is irrelevant since we will never use the ratio values without calibration).

A summary of the whole approach to particle identification is shown in Figure 6.

### Particle Tracking

To reconstruct the real trajectory of each probe, starting from their scrambled positions at each time point and their ratiometric measurement obtained in the previous steps, we built a custom algorithm inspired by the works of Jaqaman et al.^37^ The algorithm works in three steps:

#### Step 1: Frame to Frame Linking

In the first step, our goal consists in linking properly consecutive frames: this step clearly requires that the particles have already been detected and their measurements collected; thus in this step, we require the whole knowledge of all frames the experiment is made of, a frame at time t being simply the collection of all the positions of the particles, and their pH measurement at that particular time: mathematically a frame *f*_t_ at time *t* is the collection
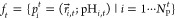
5.7where pH_*i*,*t*_ is the pH recorded by the *i*th probe in the space-time *r⃗*_*i*,*t*_.

At first, two consecutive frames—say *f*_*t*_ and *f*_*t*+1_—can have different number of particles. Since a particle present in *f*_*t*_ can disappear in *f*_*t*+1_ or, likewise, a particle can appear in frame *f*_*t*+1_^b^ we need to take into account this variable number of probes per frame, and this problem can be formulated in terms of a linear assignment problem (LAP).^68^

In this LAP, we represent probes as nodes of an abstract graph and look for its evolution: we have two collections of nodes (representing the two collections of probes in two consecutive recorded frames); we suppose in *f*_*t*_ there are *n* sensors while in *f*_*t*+1_ these are *m* such that *N* = *n* + *m*, and we aim to find out the most probable evolutions of probes in time (i.e., their trajectories). To this purpose, we introduce enlarged frames *f̂* such that
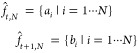
5.8and the cost for pairing two particular nodes *C*(*a*_*i*_, *b*_*j*_) = *C*_*i*,*j*_ that is arranged in a cost matrix *C* (whose entries are *C*_*i*,*j*_ where *i* is the row index and *j* is the column index). The solution of the LAP consists thus in finding the optimal assignment matrix
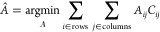
5.9where *Â* must be a binary matrix with entries 0, 1 such that
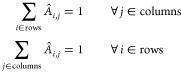
5.10thus if *Â*_*i*,*j*_ is equal to 1, then the nodes *a*_*i*_ and *b*_*j*_ are paired: roughly speaking, the optimal matrix *Â*_*i*,*j*_ can be seen as the adjacency matrix that depicts traces of the movements of the various probes within two consecutive snapshots.

The cost matrix *C*—that completely characterizes the LAP—is made of four blocks *L*, *E*, *S*, *T* as
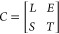
5.11where block *L* is responsible for linking a probe in frame *f*_*t*_ to the same probe in frame *f*_*t*+1_, block *E* is responsible for linking a probe in frame *f*_*t*_ to none of those in frame *f*_*t*+1_ (sensor disappearance), block *S* is responsible for linking a probe in frame *f*_*t*+1_ to none of those in frame *f*_*t*_ (sensor appearance), and block *T* is an auxiliary block that guarantees the existence of the solution for the LAP.

Let us deepen the block *L*: suppose that at frame *t*, there are *n* particles and in frame *t* + 1, there are *m* particles. The *L* block is then a *n* × *m* matrix like
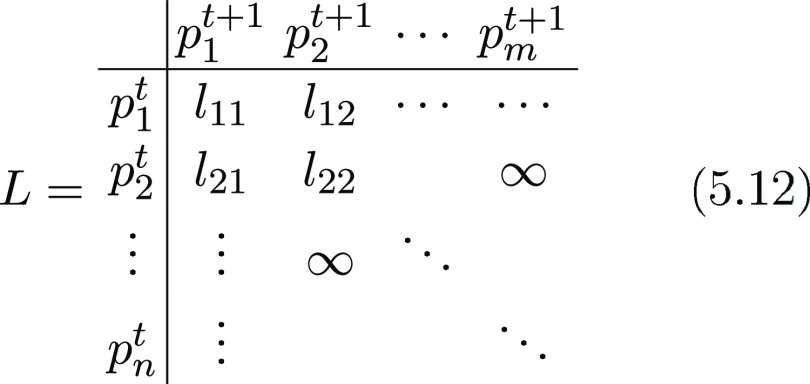
5.12where *l*_*ij*_ is the cost for linking probe *i* in frame *f*_*t*_ to probe *j* in frame *f*_*t*+1_: this cost is calculated as the modulus of their Euclidean distance (such that the ∞’s appearing in the matrix represent links which are forbidden because they would lead to a travel distance which is too large to be physically possible).

In order to construct the *E* block, we must first calculate the coefficient *b* as
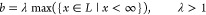
5.13

Then, the *E* block is an *n* × *n* matrix made up as follows
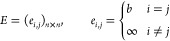
5.14

Similarly the *S* block is an *m* × *m* matrix constructed as
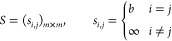
5.15as a last step, to build up the *T* block, we must first calculate the coefficient *c* as
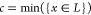
5.16such that the *T* block reads simply as an *m* × *n* matrix constructed as
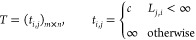
5.17thus, the *T* block is simply the *L* block but reversed in order to account for the case where all probes have been assigned and empty nodes are left to be filled.

Solving this LAP returns the best assignment matrix (whose information is stored in the *L*, *E*, *S* blocks) needed to characterize the segments that univocally connect probes from *f*_*t*_ to *f*_*t*+1_: this first step must be repeated for all pairs of adjacent frames.

Let us show an example of this procedure, starting from a fictitious cost matrix *C*: we have in the frame at time 1 only 2 particles, that is _*n*_ = 2 for *f*_1_, and in frame 2, there are 3 particles, that is _*m*_ = 3 for *f*_2_. Following the rules defined above, setting λ = 2, the cost matrix *C* would look like
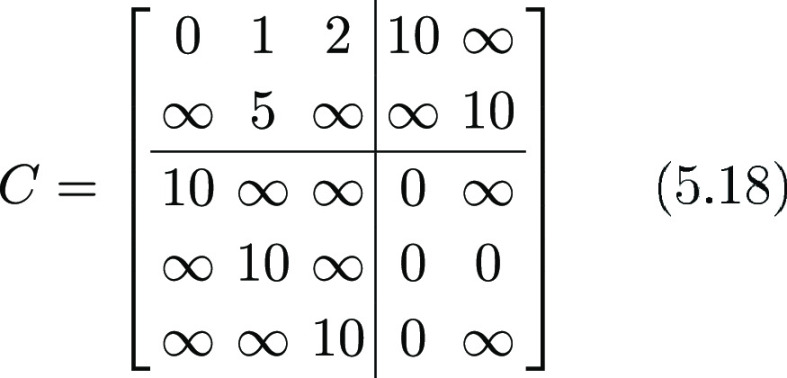
5.18

Solving the LAP for this example leads to this assignment matrix
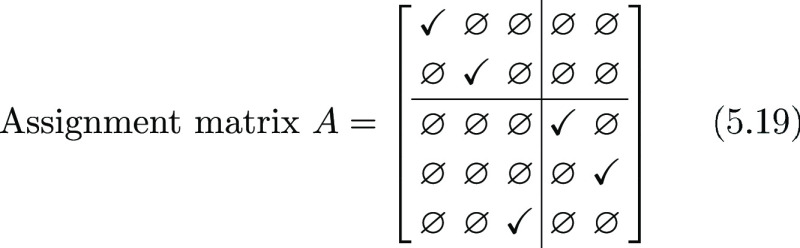
5.19

To understand which particle has been linked to which, we must simply look at the regions where the blocks *L*, *S*, *E* lie in the *C* matrix: indeed, looking at the *L* block, we can see that the first probe in frame 1 has been connected to the first probe in frame 2, the second probe in frame 1 has been connected to the second probe in frame 2, and no more assignments are present. No assignment is present in the *E* block either while one assignment on the third diagonal element of the *S* block appears, suggesting that the third particle in frame 2 is appeared in frame 2 but was missing in frame 1. So, in this case, we inferred the evolution of the probes between adjacent frames as
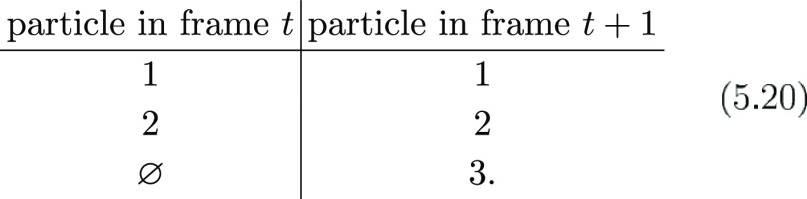
5.20

#### Step 2: Linking the Segments En Route to the Trajectory

In this step, we take all segments built in the previous step and use them to construct the longest track with no gaps inside such that if we have the segments from *t* → *t* + 1 (*S*_*t*_) and the segments from *t* + 1 → *t* + 2 (*S*_*t*+1_), grabbing from the toy example above, we can write
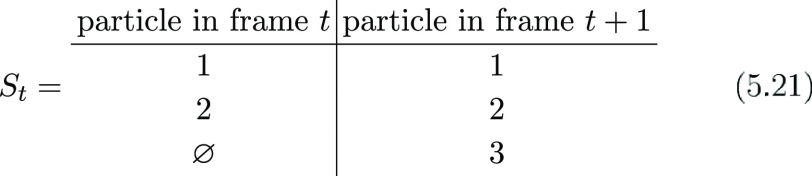
5.21
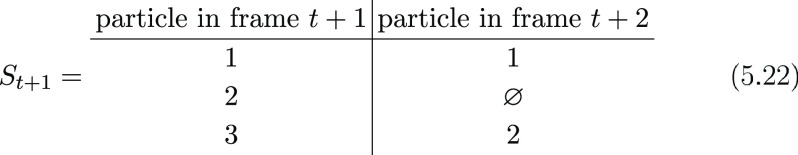
5.22such that the tracks are built by following each link from frame to frame, leading to
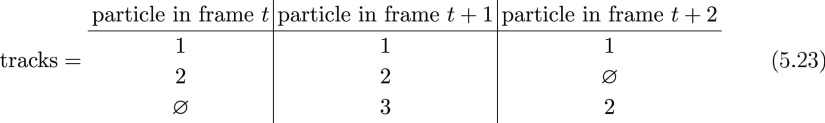
5.23

After the segments have been linked together to form the longest possible tracks, the last step is accounting for incomplete tracks.

#### Step 3: Gap Closing

This step is again formulated in terms of a LAP, but in this case, block L is built differently: still as a toy example, imagine that we have these tracks
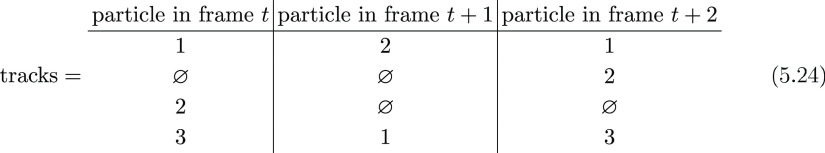
5.24where there are only two incomplete tracks: first of all, we define the collection of all tracks obtained in the first two steps as
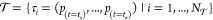
5.25and within this collection, we focus solely on the two subcollections of tracks starting at later times (w.r.t. *f*_*t*=1_) and of tracks ending before the last time. The former class reads as
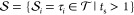
5.26while the latter, the collection of the tracks ending before the last time, reads as
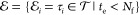
5.27where *N*_f_ is the total number of frames.

Clearly, in general, the collections  and  are built by different amounts of tracks, and we refer to these numbers, respectively, as *N*_S_ and *N*_E_.

The cost matrix C for this last LAP has unchanged structure
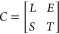
5.28although in this case the *L* block is a *N*_E_ by *N*_S_ matrix, and its elements are defined by
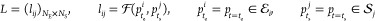
5.29where  is a function of the positions of the probes and their times of appearance
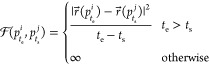
5.30

Note that, at the right-hand side of eq 5.30, the division by the temporal difference is needed in order to properly weight cost according to a Brownian motion reference model.

The remaining *S*, *E*, *T* blocks are built exactly in the same way described in step 1, and according to eqs 5.13, 5.14, 5.15, the only difference is lying in the fact that now *n* → *N*_E_ and *m* → *N*_S_.

The final step is solving this LAP characterized by the cost matrix *C* thus obtaining an assignment matrix: in this case, the only interesting block for the assignment matrix is the block corresponding to the *L* block in *C* as this block directly highlights which track is linked to which other track, allowing to consider also smaller trajectories related to missing particles. This procedure is very similar to its counterpart described in step 1; thus, we omit all gory details and we give a final example of what this step could accomplish: the probable result of applying this procedure to the fictitious tracks in (5.24) is
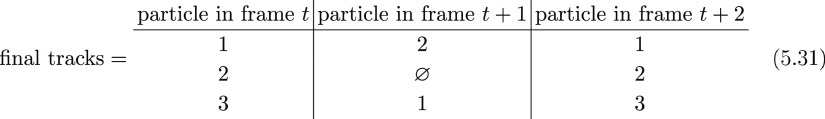
5.31if their temporal distance and physical distance allow them to be linked together, and it all depends on (5.30).

### Validation

In order to evaluate the performance of this new tracking algorithm, as a function of the density of the sensors, we extensively relied upon numerical simulations: we implemented a standard self-propelled model^38^ of *N* particles whose positions are labeled by *r*_*i*_, *i* ∈ (1, ..., *N*) and whose velocities are labeled *v*_*i*_, *i* ∈ (1, ..., *N*) and whose evolution equations read
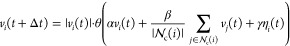
5.32
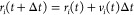
5.33where η_*i*_(*t*) is a random sample from Normal distribution,  is a set containing the topological neighbors of particle-*i*, and θ(*v*) = *v*/|*v*| is a normalization operator to transform a vector into a unit vector. The parameters α, β, γ are as follows:

α: Inertia, this coefficient tunes how strongly a particle tends to persist in its path.

β: Imitation, this coefficient dictates how strongly a particle’s trajectory tends to imitate the path of neighboring particles.

γ: Noise, this coefficient accounts for affecting a particle by Brownian randomness.

**Figure 7:**
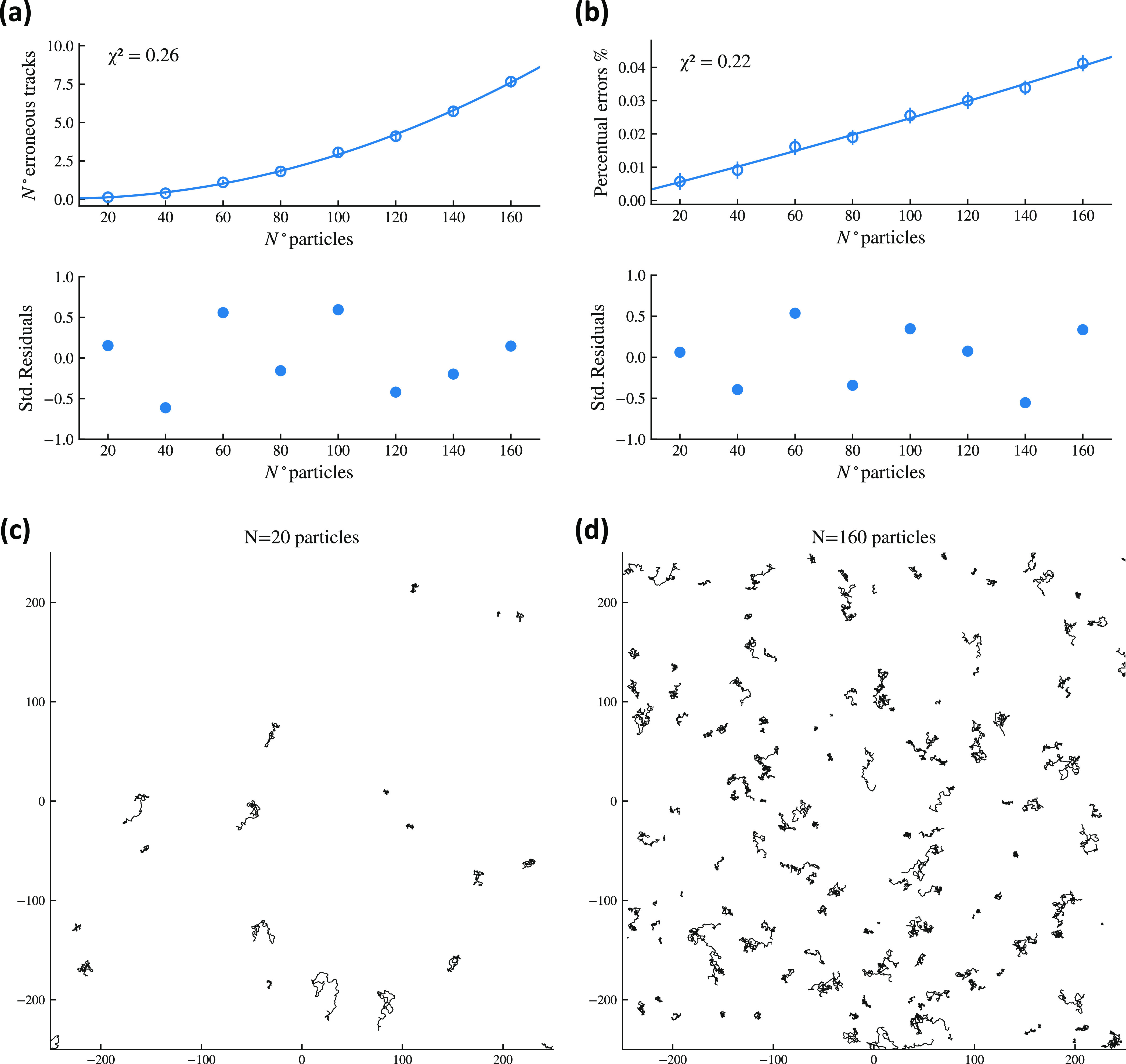
Validation of the new tracking algorithm. (a) Number of mistakes made by the algorithm in probe trajectory reconstruction as the number of sensors is increased: the parabolic shape reflects the mean field nature of the synthetic model as the number of contact among particles scales as *n*(*n* – 1)/2 ∼ *n*^2^, but the percentage of errors is kept minimal. (b) Number of mistakes made by the algorithm in probe trajectory reconstruction vs number of detected tracks (but correct and wrong): the linear growth with the density emerges as expected. (c,d) Examples of simulations of self-propelled trajectories with *N* = 20 and *N* = 160 particles, respectively.

Despite its simplicity, this elementary model leads to very interesting emergent behavior and generates quite *natural* synthetic trajectories.^69,70^ Examples can be found in Figure 7c,d.

The synthetic dataset we generated is then processed by our tracking algorithm described in the previous section: the tracking results are collected and the number of erroneous track detections are recorded. This procedure is repeated 100 times and for different particle numbers ranging from 20 → 160 with steps of 20: the results of these extensive simulation study are depicted in Figure 7.

Overall, these results show that there are very few false detections, that is, their probability is extremely low and their effect on any result derived via this tracking algorithm will be correspondingly rather marginal; moreover, if the sensor’s density is kept at reasonably values (i.e., avoiding pathological and useless crowding), wrong detections are for practical purposes zero.

## Supporting Information Available

Additional results on characterization of pH sensors based on silica microparticles, including CLSM and SEM images, size estimation, absorbance and emission spectra, estimation of number of dye molecules attached on silica microparticles; cytotoxicity assessment of the microparticles using MTT assay; and study of the intracellular localization of pH-sensors into acidic compartments of both MCF-7 and MDA-MB-231 cells (PDF)

## Notes

Availability of data and materials: The datasets used and/or analyzed during the current study are available from the corresponding author upon reasonable request.

## Footnotes

a The algorithm used is the one implemented in R version 3.6.1 (2019-07-05).

b Both the phenomena can happen for changes in focus or intensity of light, or simply the probe went out of range of detection.
