# Supplementary material for: Fully Automated Computational Approach for Precisely Measuring Organelle Acidification with Optical pH Sensors
Source: ACS Appl Mater Interfaces. 2022 Apr 11;14(16):18133–49. doi: 10.1021/acsami.2c00389 (PMC9052195; doi:10.1021/acsami.2c00389)
Supplement: Supplementary file 1 — am2c00389_si_001.pdf [file am2c00389_si_001.pdf]

Supporting information
A fully automated computational approach for precisely measuring organelle acidification with optical pH sensors

Anil Chandra,[a,*] Saumya Prasad,[a,*] Francesco Alemanno,[a,b] Maria De Luca,[c] Riccardo Rizzo,[a] Roberta Romano,[c] Giuseppe Gigli,[a,b] Cecilia Bucci,[c] Adriano Barra,[b,d,] Loretta L. del Mercato[a,**]**

[a] *Institute of Nanotechnology, National Research Council (CNR-NANOTEC), Campus Ecotekne, via Monteroni, Lecce 73100, Italy*

[b] *Dipartimento di Matematica e Fisica, Università del Salento, via Monteroni, Lecce 73100, Italy*

[c] *Dipartimento di Scienze e Tecnologie Biologiche ed Ambientali (DiSTeBa), Università del Salento, via Monteroni, Lecce 73100, Italy*

[d] *Istituto Nazionale di Fisica Nucleare, Sezione di Lecce, via Monteroni, Lecce 73100, Italy*

[*] *Equally contributing authors*

[**] *Corresponding authors*

adriano.barra@unisalento.it, loretta.delmercato@nanotec.cnr.it

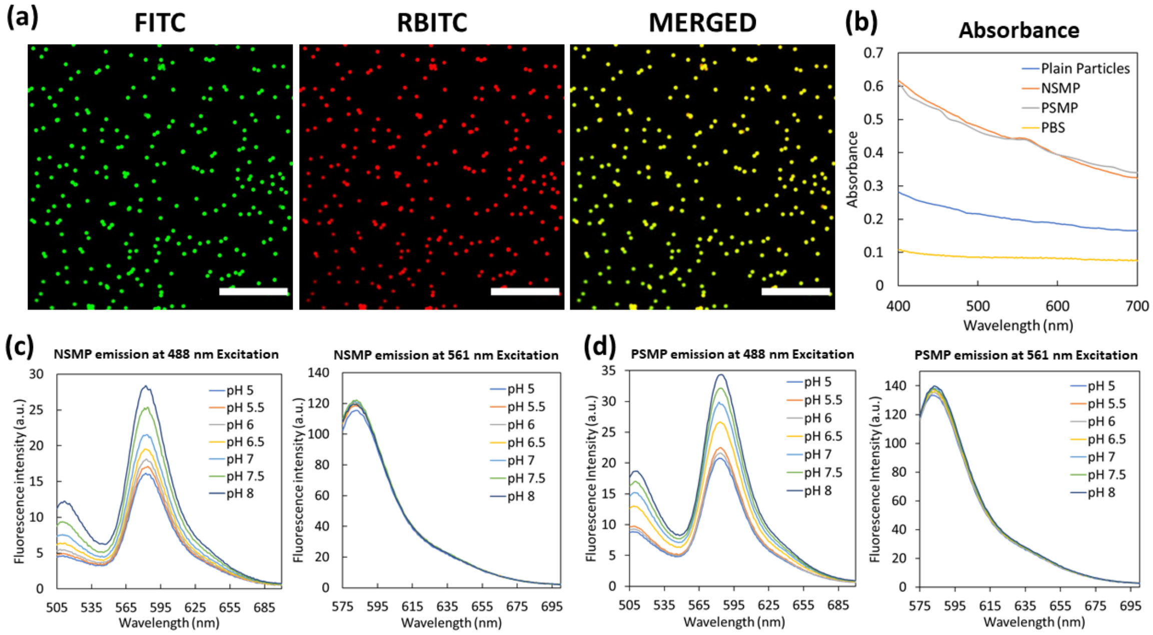

Figure S1. **(a)** Fluorescence microscopy images of NSMPs. The estimated average size of the particles is $1.13 \pm 0.05 \mu m$. Scale bars: $20 \mu m$. **(b)** Absorbance spectrum of NSMPs, PSMPs and plain silica particles. **(c)** Fluorescence emission of NSMPs and **(d)** PSMPs.

Estimation of FITC and RBITC loading in the Silica Microparticles

The absorbance spectrum of NSMPs dispersed in PBS was acquired, where the concentration of the dispersion (mg/mL) was predetermined as it was prepared using known dried NSMPs. Using the following equation, the number of particles/grams was calculated.

$$N = \frac{6 \cdot 10^{12}}{\pi \rho_s d^3},$$

where N = microspheres/gram for dry powders, ρ_s = density of solid sphere (g/cm^3), d = mean diameter (μm).

The number of particles / gram of dried solid was used to estimate particles / mL after preparation dispersion. For example, in current work, $0.3mg$ of dried microparticles is dispersed in $1mL$ PBS resulting in $3.7490x109$ particles per mL. The next step involves acquiring the absorbance spectra of the dispersion to obtain the OD at $494nm$ and $555nm$ respectively for FITC and RBITC. By using the lamber beer law ($A = \epsilon cl$) the concentration of dye molecules in the suspension can be calculated. Afterwards particles per mL can be equated with Molar concentration of the respective dye molecule. In current work following values have been used for the calculations.

Concentration of microparticles (in PBS):	$0.33mg/mL$
Absorbance at 494 nm	0.27
Absorbance at 555 nm	0.247
Molar Extinction Coefficient of FITC	$70000M^{-1}cm^{-1}$
Molar Extinction Coefficient of RBITC	$106000M^{-1}cm^{-1}$
Pathlength (cm)	0.625
Mean diameter of Microparticles	$0.93\mu m$
Density of silica microparticles	$1.9g/cm^3$

The calculated number of FITC and RBITC molecules attached per silica microparticle are $8.92 \cdot 106$ and $5.39 \cdot 106$ respectively, which is equivalent to $5.77 \cdot 10^{-15}$ and $4.80 \cdot 10^{-15}$ gram.

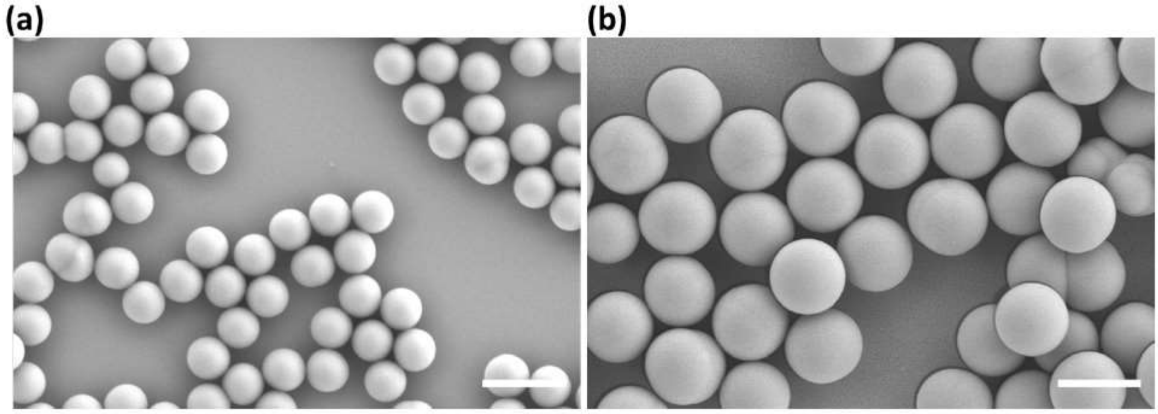

Figure S2. Size comparison of **(a)** $0.93 \pm 0.07 \mu m$ and **(b)** $1.85 \pm 0.07 \mu m$ SMPs. Scale bars: $2 \mu m$.

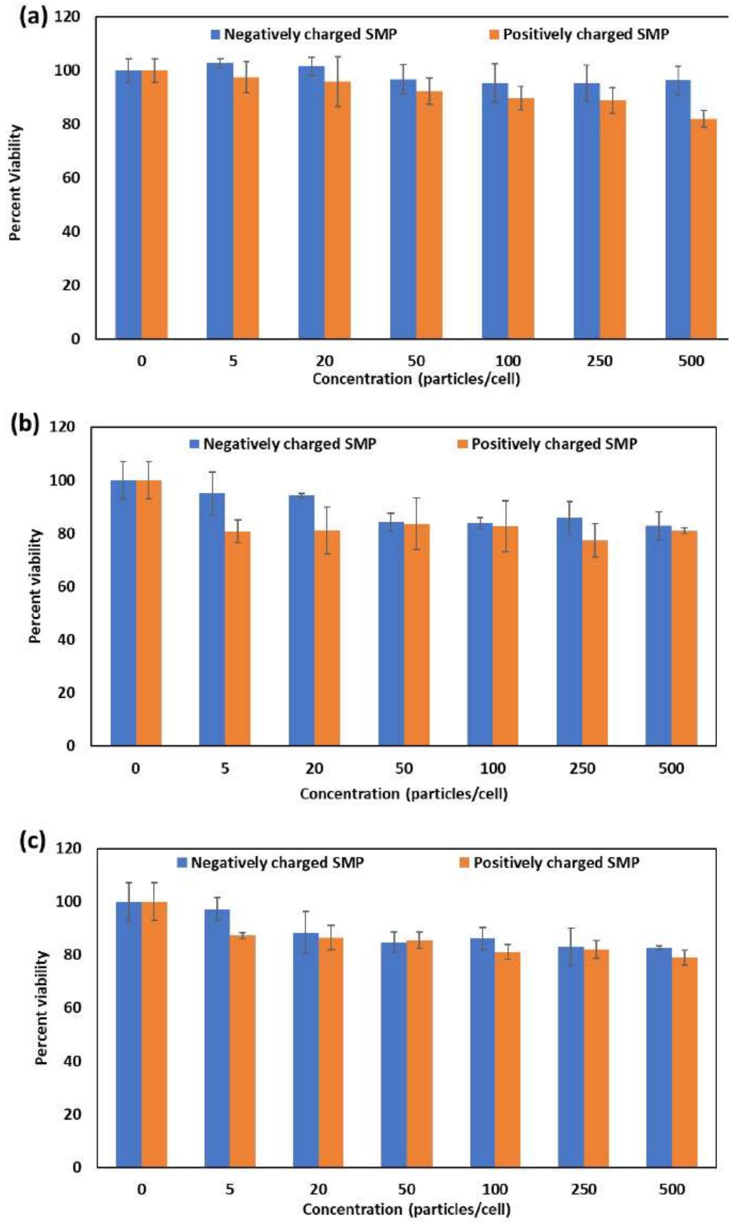

Figure S3. Cytotoxicity assessment of the microparticles using MTT assay. PSMPs and NSMPs were incubated with L3.6pl, MiaPaca and PSE cells for 24 hours. The cell seeding density for all the three cell types was 10^4 cells per well of the 96 well plate. Different concentration of microparticles was realized by taking ratio of particles per cell in the range of 5 to 500 particles per cell. No particles were added in the control well.

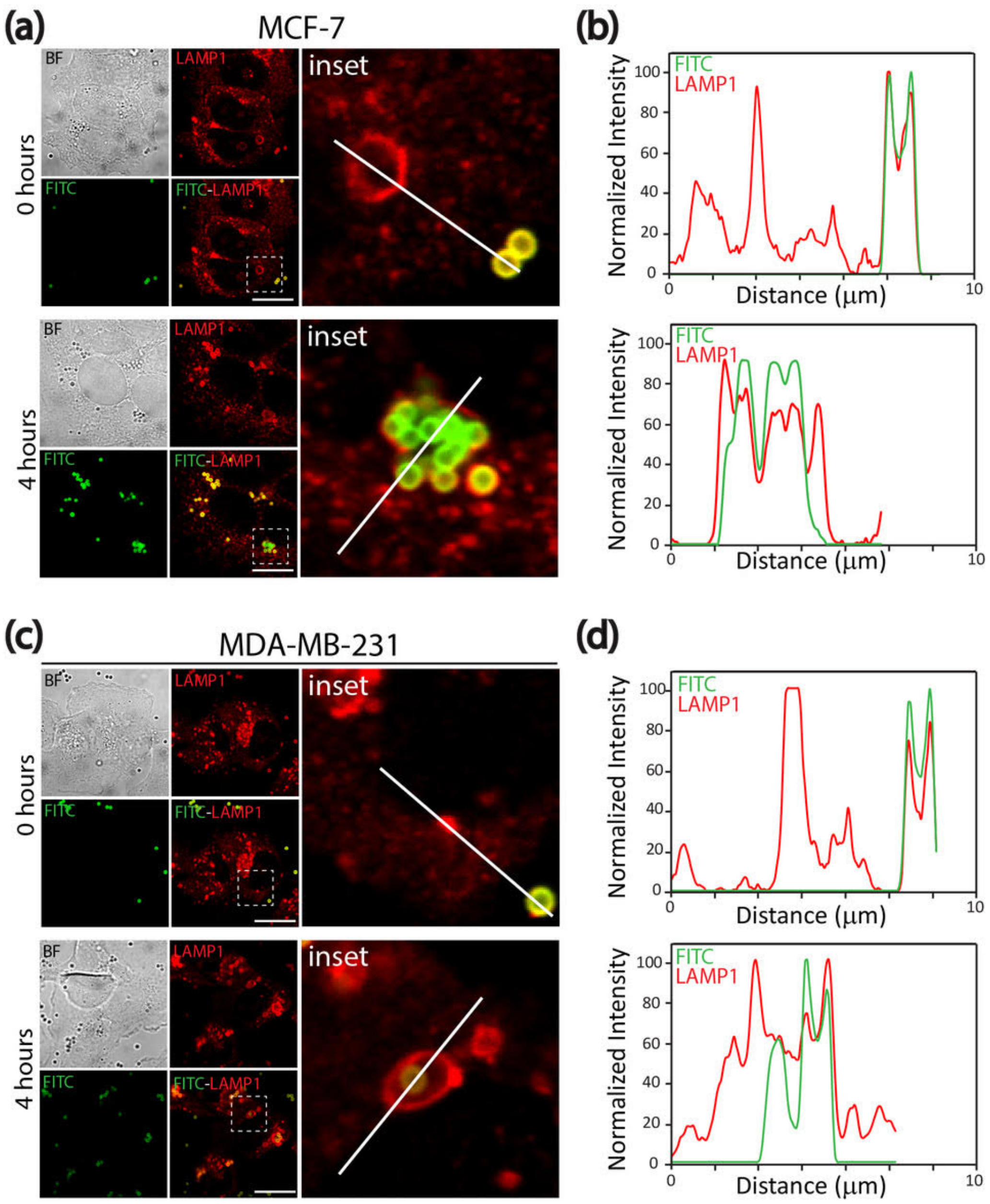

Figure S4. Localization of PSMPs in lysosomes. MCF-7 **(a)** and MDA-MB-231 **(c)** cells were exposed to PSMPs for indicated times, fixed and processed for immunofluorescence labelling: PSMPs (FITC, green), LAMP1 (Alexa 647, red false colour), BF (bright field). Scale bars, 15μm. **(b, d)** Line scan profile of the inset in (a) and (c) is reported in (b) and (d) respectively. Red line (LAMP1), green line (FITC).
